# Plant-Associated Pseudomonads

**DOI:** 10.3390/microorganisms11051216

**Published:** 2023-05-06

**Authors:** Rafael Rivilla, Jacob G. Malone

**Affiliations:** 1 Departamento de Biología, Universidad Autónoma de Madrid, 28049 Madrid, Spain; 2 Department of Molecular Microbiology, John Innes Centre, Norwich NR4 7UH, UK

Bacteria belonging to the genus *Pseudomonas* (the pseudomonads) are a group of Gammaproteobacteria that are characterized by a high metabolic versatility and adaption to different ecological niches. One of these niches, occupied by several strains and species of pseudomonads, is the plant and its microbiome, which, depending on the strain or species, may have a saprophytic or pathogenic lifestyle. Furthermore, many plant-saprophytic pseudomonads present plant growth promoting (PGP) properties and are being used in agriculture for the design of novel inoculants for plant growth and/or plant protection.

In this Special Issue, ten articles analyze different aspects of the pseudomonads–plant interaction. Both saprophytic and pathogenic pseudomonads are studied.

Regarding pathogenic pseudomonads, Moreno-Perez et al. (2021) [1] describe the HrpL regulon of the woody plant pathogen *Pseudomonas savastanoi* pv. *savastanoi*. The HrpL regulon includes the components and effectors of a type three secretion system (T3SS), which is implicated in virulence. The authors use a combination of transcriptomic analysis, bioinformatic prediction, and virulence tests to define more than 50 genes belonging to this regulon. Although many of the genes regulated by HprL belonged to the T3SS, some others may represent novel virulence factors. Another woody plant pathogen is studied in this Special Issue. In the article by Neale et al. (2021) [2], the authors use a mutagenesis approach in two patovars of *Pseudomonas syringae* to identify genes implicated in the bacterial canker disease of cherry. From a battery of mutants, they selected 18 genes affecting virulence and found that they were related to motility, type III secretion, membrane transport, amino acid synthesis, DNA repair, and primary metabolism.

Several articles in this issue deal with pseudomonads showing biocontrol ability. Ali et al. (2022) [3] show that an endophytic strain of *Pseudomonas bijeensis*, a species belonging to the *corrugata* subgroup, can control bacterial canker and gray mold pathogens of kiwifruit. They show that this bacterium produces the fungicide diacetyl-phloroglucinol (DAPG), as well as lipopeptides, and links these two traits with the antifungal and antibacterial activity of the bacterium. Yan et al. (2021) [4] analyzed the regulation of the production of the antibiotic pyoluteorin by the biocontrol strain *Pseudomonas protegens* Pf5. The production of this antibiotic is regulated by autoinduction, and two transcriptional regulators, PltR and PltZ, coordinate autoinduction. Pintado et al. (2021) [5] show that the exudate produced by the pathogenic fungus *Rosellinia necatrix* induces the expression of biofilm-related genes in a biocontrol strain of *Pseudomonas alcaligenes*. This strain shows antagonism to the fungus and feeds on its exudate. The genomic sequence of this strain is presented. They also identify a gene encoding a GGDEF-EAL protein, implicated in biofilm formation through its EAL domain. Biofilm is also the topic of the Blanco-Romero et al. (2021) article [6]. The authors of this article present an “in silico” analysis of the extracellular matrix (ECM) components of the biocontrol and model strain *Pseudomonas ogarae* F113. In this article, they describe a novel exopolysaccharide, pseudomonas acidic polysaccharide (Pap) produced by F113 and a number of other plant-associated pseudomonads. They also show that this polysaccharide, as well as an alternative type of tight adhesion pili (Tad) have co-evolved in many plant-associated pseudomonads. The presence or absence of different polysaccharides and extracellular proteins in different species of pseudomonads is also shown. Another strain with possible biocontrol ability is *Pseudomonas fluorescens* WH6. This strain produces the non-proteinogenic amino acid 4-formylaminooxyvinylglycine (FVG), a secondary metabolite with antibacterial and pre-emergent herbicidal activities. Manning and Trippe (2021) [7] generated KO mutants in *gvg* genes implicated in the regulation of FVG production and found that many genes were regulated by this system. They also showed that in KO mutants that did not produce FVG, resources were mobilized to increase phenotypes involved in rhizocompetence, including motility, biofilm formation, and denitrification. Hoffmann et al. (2021) [8] use a biocontrol strain of *Pseudomonas simiae* to show that the order of inoculation in the wheat of this strain and two fungal pathogens had an effect in the outcome of the plant–bacteria–fungi interaction and especially in the production of mycotoxins.

Finally, two articles deal with the environmental adaption of plant-associated pseudomonads. Lalaouna et al. (2021) [9] show the importance of the Gac system and its associated Rsm small RNAs in the adaption of *Pseudomonas brassicacearum* to nutrient-poor environments. They also show the linkage of the Rsm pathway to the stringent response and to the levels of the messenger molecule c-di-GMP. The article by Breitkreutz et al. (2021) [10] analyzes the influence of drought and plant community composition in the diversity of pseudomonad genotypes in grasslands. Their results suggest that the *Pseudomonas* community quickly responds to drought in terms of structure and function, while the extent of the response is affected by the plant community composition. These functional changes are trait-specific.

This collection of 2021–2022 articles reflects the state of the art of research in pseudomonads–plant interactions, and shows the relevance that genomic techniques, such as sequencing, transcriptomics, and genome-wide mutagenesis, as well as bioinformatic analysis, are having in this research. It also highlights the diversity of lifestyles of pseudomonads when interacting with plants and their potential application in agricultural biotechnology.
